# Prevalence of ABH(O) Blood Group Secretors Among Indigenous Population of Shillong: A Cross-Sectional Study

**DOI:** 10.7759/cureus.73266

**Published:** 2024-11-08

**Authors:** Prabal Das, Daunipaia Slong, Amarantha D Ropmay, Amar J Patowary, Lokesh Ravi V Naidu, Bhaskar Mukherjee

**Affiliations:** 1 Forensic Medicine, North Eastern Indira Gandhi Regional Institute of Health and Medical Sciences, Shillong, IND

**Keywords:** abh(o), agglutination, anti-serum, blood groups, evidence, saliva, secretors, shillong

## Abstract

Background

ABH antigens on the surface of red blood cells form Type A, B, AB, and O blood groups. These antigens are also found in body secretions like serum, gastric juices, ovarian cyst fluid, semen, amniotic fluid, sweat, urine, tears, bile, and saliva (except cerebrospinal fluid). People who have these antigens in their body fluids are called secretors.

Purpose

This study was conducted to find out the prevalence of salivary secretor status among the adult local indigenous population of Shillong, India.

Method

Unstimulated saliva was collected and processed from 250 apparently healthy adults, determined its secretor status using the absorption-inhibition method, and then analyzed using the Chi-square test to find its association with age, sex, tribe, and blood groups.

Results

The study found a prevalence of salivary secretor status of 57.2% (n=143), with a slight female predominance of 57.7% (n=64) over males (56.8%, n=79). The highest percentage was found in individuals aged 71-80 (68.8%, n=16), while the least in people aged 18-30 (48.2%, n=40). Khasi tribes had the highest incidence (64.2%, n=88), followed by Garo (55%, n=22), and least in Jaintia tribes (45.2%, n=33). O-blood group individuals had the highest percentage (71%, n=22), while AB-blood group individuals had the least (46%, n=23). We observed a statistically significant correlation of salivary secretor status with the blood group and tribe of the individuals but not with their sex and age.

Conclusion

Saliva can be found on various items in medicolegal cases like robberies, rapes, and hangings, but blood stains may not always be present. Saliva can be used to determine blood groups, potentially aiding in resolving claims of parentage, immigration, kidnapped children, disputed paternity, or identifying victims in mass disasters. ABO(H) determination from body fluids, especially saliva, has been used in forensic investigations before DNA technology. Currently, this can be an initial screening method because DNA methodology is not often available in laboratories in India.

## Introduction

Identification is a unique attribute that identifies a person and is required in various situations, including civil matters, criminal cases, and humanitarian purposes. It can be absolute or partial and is necessary in living or dead persons, decomposing bodies, burned bodies, disfigured bodies, or when a body part is discovered [1,2]. In forensic medicine, identification has evolved into an art of science involving medical professionals and police investigators to determine a person's identity [3].

One of the methods to identify individuals is through blood grouping, which is based on the presence and absence of antigens on Red Blood Cells (RBCs). There are several blood group systems identified over the years, with ABO being the most important due to the prominence of anti A and anti B antibody six months after birth [4]. The ABO blood grouping system, discovered by Landsteiner K in 1900 [5-7], recognized 4 blood group types namely A, B, AB, and O based on the type of A, B and H antigens found on the surface of red blood cells. These antigens, developed from their H-substance precursor, can only partially transform into A or B and are present in most bodily fluids as soluble compounds, except cerebrospinal fluid [6-8]. A person’s ability to secrete ABH antigens in body fluids determines his or her status as a secretor or non-secretor. Saliva stain is frequently found in crime scenes either on items such as food, toothpick envelop flaps, etc, or on the dead body from bite marks, etc. From these stains, the ABO blood group can be detected with a high degree of accuracy using the absorption elution method or absorption inhibition method [1,3,5,6], with the latter being simpler or easier to perform [3,9,10].

Several studies on the salivary secretor status of ABH(O) blood group antigens have been conducted worldwide and in other parts of India. In North East India, one such study was done by Devi et al. [11] among the Manipuri population. However, to the best of our knowledge, no such study has been conducted in Meghalaya. Studies also show there is geographic and racial variation in the proportion of secretors among the global population [11-14]. Therefore, the present study is conducted to find out the prevalence of ABH(O) blood group secretors and non-secretors, using the absorption inhibition method, among the indigenous population of Shillong comprising of Khasi, Jaintia, and Garo tribes.

## Materials and methods

This cross-sectional study was conducted in the Department of Forensic Medicine of a Tertiary Care Hospital in Shillong, Meghalaya (India) from 26th April 2021 to 31st October 2022 after getting approval from the Institutional Ethics Committee (IEC) (NEIGR/IEC/M14/T9/2021 dated 26th April 2021). A total of 250 saliva samples were collected, using a convenient sampling method, from local indigenous healthy adult residents of Shillong, comprising of Khasi, Jaintia, and Garo tribes, who gave consent for the study. Participants provided information like age, sex, tribe, and blood group. Date and time of collection were also noted. Exclusion criteria include (i) People with a history of blood transfusion and bone marrow transplantation, (ii) People with malignancies like leukemia, which leads to weakening or loss of blood group antigens on cells, (iii) People with any other haematological diseases/disorders/syndromes, (iv) People with a history of gram-negative septicaemia, intestinal obstruction and colon/rectum carcinoma leading to acquired ‘B’ antigen like activity.

Collection and processing of saliva

After proper rinsing of the mouth with water, about 2 ml of saliva sample from each participant was collected in a sterile plastic container with a unique code for laboratory investigation. The sample was transferred to a test tube, which was then sealed and then denatured in boiling water for 10 minutes [5,7,12], cooled for the next 10 minutes at room temperature [7], and centrifuged at 3000 rpm for the next 10 minutes [3,7]. The supernatant was diluted with an equal volume of normal saline and divided into three test tubes (1A, 1B, and 1H). Controls were made by adding normal saline in three different test tubes (2A, 2B, 2H). The samples were immediately tested for secretory status by the absorption-inhibition method.

Preparation of anti-sera and then mixing of anti-sera with saliva

In separate test tubes, saline solution was used to dilute polyclonal A and B anti-sera, each at a 1:10 ratio [12,13]. Commercially available Anti H was used to determine the secretor status of the participants. The diluted saliva in test tubes 1A, 1B, and 1H was then mixed with one drop of diluted Anti-A, Anti-B and Anti-H, respectively. Similarly, each of the three diluted anti-sera was mixed with the control in test tubes 2A, 2B and 2H. The test and control samples were allowed to stand for 10 minutes at room temperature [3,12].

Adding indicator cells

After 10 minutes, one drop of thrice-washed 3-5% red cell suspension, i.e., pooled cells of A, B and O blood groups (prepared and provided by the Department of Transfusion Medicine and Blood Centre of the same Tertiary Care Hospital) were added to each test tube as indicator cells as shown in Table 1. The test tubes were mixed thoroughly by gentle shaking and then kept at room temperature for about one hour [10].

**Table 1:** Table showing the procedure of adding indicator cells to respective test tubes according to labels

	Test tube labels	Antisera	Indicator cells
Test samples (with saliva)	Test tube 1A	Anti-A	A-cells
	Test tube 1B	Anti-B	B-cells
	Test tube 1H	Anti-H	O-cells
Control samples (with normal saline)	Test tube 2A	Anti-A	A-cells
	Test tube 2B	Anti-B	B-cells
	Test tube 2H	Anti-H	O-cells

Interpretation of results

**Figure 1:**
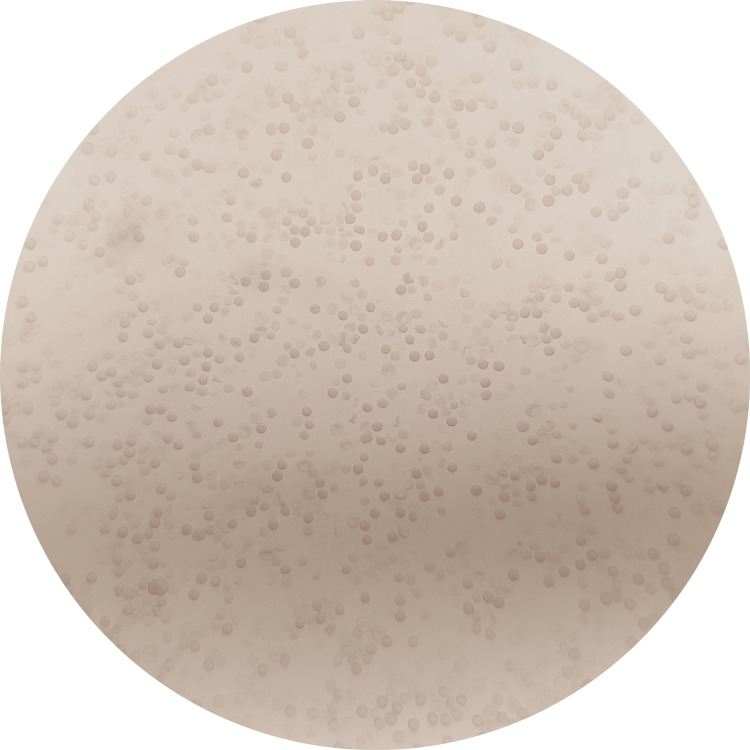
Microscopic view of all free cells under 10X (no agglutination) The test samples were considered positive if no agglutination was observed, indicating that an antigen-antibody reaction had occurred between saliva and anti-sera and that there was no antibody left for red blood cells (indicator cells) to react.

**Figure 2:**
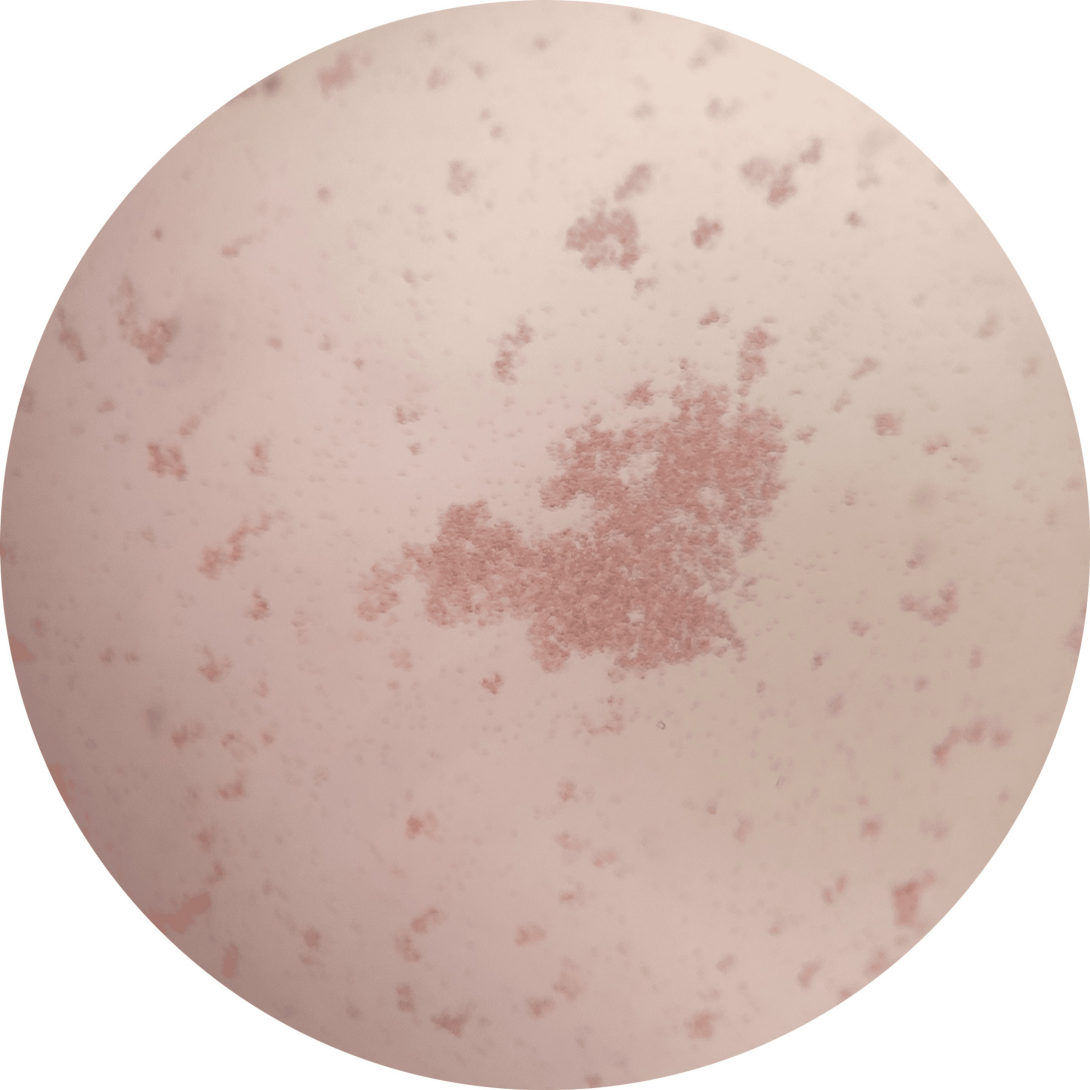
Microscopic view of small to large masses of agglutination under 10X (positive agglutination) The test samples were considered negative if agglutinationwas* observed*, indicating that an antigen-antibody reaction had not occurred between saliva and anti-sera and that there was an antibody left for red blood cells (indicator cells) to react.

The samples were examined for agglutination both macro and microscopically (Figures 1-2). The absence of agglutination in test samples was taken as a positive result for the absorption-inhibition method [5-7,11,12]. To validate the result, it was compared with the positive agglutination in controls. The test samples were considered positive if no agglutination was observed, indicating that an antigen-antibody reaction had occurred between saliva and anti-sera and that there was no antibody left for red blood cells to react, indicating the presence of blood group [1,3,5,7,9,13].

Statistical analysis

Data was analyzed using IBM Statistical Package for Social Sciences (SPSS) Statistics Software for Windows, version 28.0.1.1 (IBM Corp., Armonk, NY). We conducted a Chi-square test to determine the association of salivary secretor status with independent variables like sex, age, ethnicity/tribe, and blood groups. A p-value of less than 0.05 at a 95% confidence interval (CI) is considered statistically significant.

## Results

Out of 250 subjects, 139 (55.6%) were males and 111 (44.4%) females, with 137 (54.8%) Khasi, 73 (29.2%) Jaintia, and 40 (16%) Garo. One hundred and sixteen (46.4%) had B blood group, 53 (21.2%) A blood group, 50 (20%) AB blood group, and the remaining 31 (12.4%) O blood group.

As depicted in Table 2, there were 143 (57.2%) secretors and 107 (42.8%) non-secretors in our study population. Among the 143 secretors, 79 (56.8%) were males and 64 (57.7%) were females.

**Table 2:** Table showing the frequencies (n) of secretors and non-secretors in the study group

	Frequencies (n)	Percentages (%)
Secretors	143	57.2%
Non-Secretors	107	42.8%
Total (N)	250	100%

However, the association between secretor status and the sex of the individuals was statistically insignificant (X^2^(1, *N* = 250) = 0.017, p = .896), as shown in Table 3. Further analysis showed that despite having the maximum number of participants of all the age groups, 18-30 years (n=83, 33.2%) have the least frequency of secretors (n=40, 48.2%).

**Table 3:** Table showing the frequencies (n) of secretors and non-secretors in relation to the sex of the study group X^2^(1, *N* = 250) = 0.017, p = .896 (Statistically insignificant)

	Sex of the study participants					
	Males		Females		Total [N]	
	n	%	n	%	N	%
Secretors	79	56.8%	64	57.7%	143	57.2%
Non-secretors	60	43.2%	47	42.3%	107	42.8%
Total [N]	139		111		250	100%
	55.6%		44.4%			

The highest frequency of secretors was found in the age group of 71-80 years (n=11, 68.8%). However, the association between secretor status and age of the individuals was also statistically insignificant (X^2^(6, *N* = 250) = 5.790, p = .447), as shown in Table 4.

**Table 4:** Table showing frequencies (n) of secretors and non-secretors in relation to age group of the individuals X^2^(6, *N* = 250) = 5.790, p = .447 (Statistically insignificant)

	Age groups of the study participants															
	18 to 30		31 to 40		41 to 50		51 to 60		61 to 70		71 to 80		> 81		Total [N]	
	n	%	n	%	n	%	n	%	n	%	n	%	n	%	N	%
Secretors	40	48.2	30	60	25	67.6	15	53.6	17	60.7	11	68.8	5	62.5	143	57.2
Non-secretors	43	51.8	20	40	12	32.4	13	46.4	11	39.3	5	31.3	3	37.5	107	42.8
Total	83	33.2	50	20	37	14.8	28	11.2	28	11.2	16	6.4	8	3.2	250	100%

Our study also tried to determine the distribution of the secretors among the three major tribes of Meghalaya. It was observed that the Khasi tribe (n=88, 64.2%) had the highest frequency of secretors, followed by Garo (n=22, 55%) and Jaintia tribes (n=33, 45.2%). The association between secretor status and the tribe was found to be statistically significant (X^2^(2, *N* = 250) = 7.137, p = .028) as shown in Table 5.

**Table 5:** Table showing the frequencies (n) of secretors and non-secretors in relation to tribes of the individuals X^2^(2, *N* = 250) = 7.137, p = .028 (Statistically significant)

	Tribes of the study participants							
	Khasi		Jaintia		Garo		Total (N)	
	n	%	n	%	n	%	N	%
Secretors	88	64.2%	33	45.2%	22	55.0%	143	57.2%
Non-secretors	49	35.8%	40	54.8%	18	45.0%	107	42.8%
Total (N)	137		73		40		250	100%
	54.8%		29.2%		16%			

Although having the lowest number of participants (n=31, 12.4%), people with the O-blood group have the highest frequency of secretors (n=22, 71%), while the AB blood group (n=23, 46%) has the lowest frequency. The association of secretor status with the blood group of the individuals was found to be statistically significant (X^2^(3, *N* = 250) = 8.696, p = .033), as shown in Table 6.

**Table 6:** Table showing the frequencies (n) of secretors and non-secretors in relation to blood groups of the individuals X^2^(3, *N* = 250) = 8.696, p = .033 (Statistically significant)

	Blood Groups of the study participants									
	A		B		AB		O		Total (N)	
	n	%	n	%	n	%	n	%	N	%
Secretors	25	47.2%	73	62.9%	23	46.0%	22	71.0%	143	57.2%
Non-secretors	28	52.8%	43	37.1%	27	54.0%	9	29.0%	107	42.8%
Total (N)	53		116		50		31		250	100%
	21.2%		46.4%		20.0%		12.4%			

## Discussion

The proportion of secretors (57.2%, n=143) among the population in our study bores similarity to studies conducted in Dhaka [8], Karachi [9], Kerala [14], and Maharashtra [15] who reported secretor status in their population ranging from 58.6% to 64.47%. However, numerous studies [1,5-7,10,16-23] have reported a higher prevalence of ABO blood group antigens in the saliva of their study groups, with results varying from 65 to 95%. Some studies [12,13,24] have reported 100% secretor positivity but with a small sample size. However, two studies, both in 2015, one by Devi et al. [11] and the other by Muddathir et al. [25], showed a higher prevalence of non-secretors than secretors in their study groups. While the former study found 50.5% non-secretors on 400 Manipuri individuals, the latter study found 68.2% non-secretors on 566 Sudanese people. These variations in the results reflect the significance of racial [11], geographical, and ethnic factors [25] in determining the secretor status of an individual.

Our study also found that the secretor status of an individual does not have any statistically significant association with a person’s sex, even though there is a slight difference in the number of secretors among females (57.7%, n=64) in comparison to the male population (56.8%, n=79). These findings are in agreement with Kumar et al. [1], Tejasvi et al. [5], Saboor et al. [9], Woike et al. [16], and Sherwani et al. [21]. Other studies [18,19] also reported statistically non-significant association between secretor status and the sex of a person, but with a minimal male predominance. However, in 2020, BoKhedher et al. [6] and Kuttath and Nambiar [14] found a significant association of secretor status with the sex of the individuals. The former reported 73.53% female secretors, while the latter reported 75% female secretors. The latter explained the difference in their finding in their data could be because female donors comprised only 14.5% of the total samples.

There is limited literature on the association between salivary secretor status and age, possibly due to the fact that salivary secretory status is not affected by age [20,26-28]. To compare with our result, there is one study conducted by Woike et al. [16], who observed that the highest frequency of secretors (75.8%) was in the age group of 41-50 years and the least (33.3%) in the age-group of 51-60 years. They have mentioned the fact that it was difficult to draw any relevant conclusion on the low secretor status in the 51- 60 years group, because of low group sample size (six out of 1001, 0.6%]. This is in contrast to the present study where the highest frequency of secretors (68.8%, n=11 ) is found in the age group of 71-80 years and the lowest (48.2%, n=40) in the age group of 18-30. However, both studies could not establish a statistically significant relationship between secretory status and age group of the individual.

To the best of our knowledge, no research has been done on the secretor status of ABO blood grouping in the saliva of the Khasis, Jaintias, and Garos, the indigenous people of Shillong, Meghalaya. However, research has been done on other tribes from different regions of India, which includes the tribes from Manipur [11], Gujarat [17], and Punjab [22]. Our study found that the Khasi tribe had the highest incidence of secretors at 64.2% (n=88), followed by the Garo and Jaintia tribes at 55% (n=22) and 45.2% (n=33), respectively. Interestingly, the Jaintia tribe had a higher incidence of non-secretors at 54.8% (n=40), which was not found in the other two tribes. An unequal distribution of cases according to tribes may be a reason for such an outcome. The association between secretor status and tribe was statistically significant.

This study revealed a statistical relationship between salivary secretor status and blood group, with the highest percentage in individuals with O blood group (71.0%, n=22) and the lowest in the AB blood group (46.0%, n=23). These results are consistent with other studies [11,19] which also discovered a statistically significant correlation between the blood groups and the secretor status of individuals, with the O-blood group having the highest prevalence of secretors and the AB-blood group having the lowest. However, a study by Karpoor et al. [18] showed no association between secretor status and blood group, even though they found more secretors among the O blood group and lowest in the AB blood group. In contrast to our findings, there are studies those reported the highest secretors among the other blood groups like A [1,6,14], B [9,10,29], AB [7] and lowest in A [10] and B [7], AB [1,6,14].

Limitations of our study

(i) The study did not include participants below 18 years age, (ii) The study did not include diseased individuals, so the association of salivary secretor status with any disease has not been found, (iii) The study included only fresh saliva samples, not old dried ones, (iv) The technique used for determining the salivary secretor status was absorption-inhibition, which is easier and simpler, but few authors recommend the absorption-elution technique to be more sensitive, (v) The study did not compare the salivary ABH(O) antigens with those in blood, (vi) The study did not find association between Rh-antigen with salivary secretor status, (vii) The study did not find the effect of environmental factors on the detectability of blood group antigens from saliva.

Recommendation(s)

In order to address all the limitations, drawbacks, and issues encountered in the present study, it is recommended that future studies might be conducted in the following directions (i) A study with a larger population selected randomly with no age limit, (ii) A comparison study between absorption-inhibition and absorption-elution methods, (iii) The association of salivary secretor status with Rh-antigen can be analysed, (iv)The association of salivary secretor status with diseases can also be analysed, (v) The ABH(O) antigens detected from saliva can be compared with those in blood using standard slide or tube agglutination methods, (vii) The effect of environmental factors on the detectability of blood group antigens from saliva can also be analysed, (viii) The salivary secretor status can also be established using dried and old saliva stains.

## Conclusions

The study found that salivary secretor status was prevalent in 57.2% of individuals, with a statistically significant correlation with blood group and tribe of the individuals but not with their sex and age. The technique may not be as helpful as with current DNA technologies; however, it has many advantages, considering the lack of infrastructure for DNA laboratories in India. It could provide an alternate non-invasive method for initial screening of blood investigations from saliva samples found at crime scenes on cigarette buds, high-touch materials, and the body of victims, even if it is old or dried, helping to find secretors, which can be matched with suspects. In addition to blood grouping, saliva samples can also be used for deoxyribonucleic acid typing, sex determination, and bite mark analysis.

## References

[1] Jha JK, Biradar V, Mular A, Reddy SG (2018). Efficacy of saliva in determination of ABO blood groups in humans. IJSR.

[2] Aggrawal A (2021). Textbook of Forensic Medicine and Toxicology.

[3] Metgud R, Khajuria N, Mamta Mamta, Ramesh G (2016). Evaluation of the secretor status of ABO blood group antigens in saliva among southern Rajasthan population using absorption inhibition method. J Clin Diagn Res.

[4] Farhud DD, Yeganeh MZ (2013). A brief history of human blood groups. IJPH.

[5] Tejasvi ML, Bukkya JL, Rao PR, Bhayya H (2021). Evaluation of the secretor status of ABO blood group antigens in saliva using absorption inhibition method. Glob Med Genet.

[6] BoKhedher R, Al-Shalawi H, Babikir M, Mohsen DM (2020). Effect of gender and ABO blood groups on frequency of ABH antigens secretor status. Arab J Med Sci.

[7] Ullah N, Khan H, Zeb MA, Khan F, Khan M (2018). Frequency of ABH secretors and non-secretors among the students at Institute of Paramedical Sciences, Khyber Medical Univeristy, Pakistan. Indo Am J P Sci.

[8] Akhter S, Kibria G, Akhter N, Habibullah M, Islam S, Zakariah M (1970). ABO and Lewis blood grouping with ABH secretor and non-secretor status: a cross sectional study in Dhaka. Faridpur Med Coll J.

[9] Saboor M, Ullah A, Qamar K, Mir A, Moinuddin Moinuddin (2014). Frequency of ABH secretors and non secretors: a cross sectional study in Karachi. Pak J Med Sci.

[10] Onwuka KC, Tijjani BM, Gwarzo-Kuliya A, Samaila AA, Borodo MM (2012). Relationship between ABO Blood Group and ABH secretor status in Kano, North-western Nigeria. Nigeria J Med.

[11] Devi AS, Meera Th, Singh KhP, Nabachandra H, Shah I (2015). Secretors in Manipuri population: a study. J Ind Acad Forensic Med.

[12] Velani PR, Shah P, Lakade L (2018). Determination of ABO blood groups and Rh typing from dry salivary samples. Int J Clin Pediatr Dent.

[13] Thrumiaya T, Gayathri R, Vishnu Priya V (2017). Efficacy and accuracy of ABO blood group determination from saliva. J Adv Pharm Edu Res.

[14] Kuttath V, Nambiar SB (2020). Prevalence of secretor antigen and association with blood group among blood donors attending a tertiary care blood bank in Kerala. Int J Sci Res.

[15] Das SR, Kumar N, Bhatacharjee PN, Sastry DB (1961). Blood groups (ABO, M-N and Rh), ABH secretion, sickle-cell, P.T.C. taste, and colour blindness in the mahar of Nagpur. J Royal Anthropol Ins Great Britain Ireland.

[16] Woike P, Iyengar S, Sharma DC, Gaur R (2017). Frequency of ABH secretors/non secretors and its clinical significance: a cross sectional study in Gwalior. J Dent Med Sci.

[17] Vyas GN, Bhatia HM, Sukumaran PK, Balkrishnan V, Sanghvi LD (1962). Study of blood groups, abnormal hemoglobins and other genetical characters in some tribes of Gujarat. Am J Phys Anthropol.

[18] Karpoor C, Shettar SavithaS, Jatti Vijayakumar B, Kulkarni V (2010). Study of secretors and non-secretors in normal healthy population-Its forensic implication in human identification. Ind J Forensic Med Toxicol.

[19] Igbeneghu C, Olisekodiaka JM, Alabi T, Onuegbu JA, Oseni BA, Odaibo A (2015). ABH secretors status in Osogbo, Southwestern Nigeria. J Life Sci.

[20] Yadgari Y, Dawlaty B (2019). Study of secretory status of ABO blood groups antigens and Rh typing in dried salivary samples of normal individuals. IJRDO J Health Sci Nur.

[21] Sherwani SK, Ahmad H, Ahmad T, Hussain T, Akbar S, Zaidi SA, Kazmi SU (2014). Status of secretor and non-secretor with respect to ABO Blood group system in
young population in Karachi-Pakistan. World J Med Sci.

[22] Seth S (1968). A study of the A-1A-2BO blood group system and ABO(H) secretion in six endogamous groups of Punjab. Am J Phys Anthropol.

[23] Motghare P, Kale L, S Bedia A, Charde S (2011). Efficacy and Accuracy of ABO Blood Group Determination from Saliva. JIAOMR.

[24] Ruth MMA, Purnadianti M, Marini M (2020). Blood group analysis from cigarette butts by absorption inhibition method: An experimental study. J Int Oral Health.

[25] Muddathir AR, Fadl-Elmula IM, Abdelgadir RE, Bashir ASI (2015). Frequencies of secretors and non-secretors of ABH group substances among Sudanese population. American Journal of Research Communication.

[26] Badiye A, Kapoor N, Badiye V (2014). A study on salivary hemagglutinins in a Central Indian population. EJFS.

[27] Blackwell CC, James VS, Davidson S, Wyld R, Brettle RP, Robertson RJ, Weir DM (1991). Secretor status and heterosexual transmission of HIV. BMJ.

[28] Emeribe AO, Igweagu CA, Osim EE (1992). ABH secretor status in saliva of Calabar Municipality residents. East Afr Med J.

[29] Salih KM, Jouda JAK, Al-Jaff SHK, El-Haboby BT, Shaker EW (2015). Frequency of ABO blood groups and secretor status and their relation with dental decay in sections of students and employees of Al-Mustansiriyiah University, Iraq. World J Exp Biosci.

